# Stream Microbial Community Structured by Trace Elements, Headwater Dispersal, and Large Reservoirs in Sub-Alpine and Urban Ecosystems

**DOI:** 10.3389/fmicb.2020.491425

**Published:** 2020-11-26

**Authors:** Erin Fleming Jones, Natasha Griffin, Julia E. Kelso, Gregory T. Carling, Michelle A. Baker, Zachary T. Aanderud

**Affiliations:** ^1^Department of Plant and Wildlife Sciences, Brigham Young University, Provo, UT, United States; ^2^Department of Biology and the Ecology Center, Utah State University, Logan, UT, United States; ^3^Department of Geological Sciences, Brigham Young University, Provo, UT, United States

**Keywords:** bacterioplankton, dispersal, water chemistry, species sorting, urban, reservoir, community composition

## Abstract

Stream bacterioplankton communities, a crucial component of aquatic ecosystems and surface water quality, are shaped by environmental selection (i.e., changes in taxa abundance associated with more or less favorable abiotic conditions) and passive dispersal (i.e., organisms’ abundance and distribution is a function of the movement of the water). These processes are a function of hydrologic conditions such as residence time and water chemistry, which are mediated by human infrastructure. To quantify the role of environmental conditions, dispersal, and human infrastructure (dams) on stream bacterioplankton, we measured bacterioplankton community composition in rivers from sub-alpine to urban environments in three watersheds (Utah, United States) across three seasons. Of the 53 environmental parameters measured (including physicochemical parameters, solute concentrations, and catchment characteristics), trace element concentrations explained the most variability in bacterioplankton community composition using Redundancy Analysis ordination. Trace elements may correlate with bacterioplankton due to the commonality in source of water and microorganisms, and/or environmental selection creating more or less favorable conditions for bacteria. Bacterioplankton community diversity decreased downstream along parts of the stream continuum but was disrupted where large reservoirs increased water residence time by orders of magnitude, potentially indicating a shift in the relative importance of environmental selection and dispersal at these sites. Reservoirs also had substantial effects on community composition, dissimilarity (Bray-Curtis distance) and species interactions as indicated by co-occurrence networks. Communities downstream of reservoirs were enriched with anaerobic Sporichthyaceae, methanotrophic Methylococcaceae, and iron-transforming Acidimicrobiales, suggesting alternative metabolic pathways became active in the hypolimnion of large reservoirs. Our results identify that human activity affects river microbial communities, with potential impacts on water quality through modified biogeochemical cycling.

## Introduction

Bacterioplankton, the portion of stream microbial communities suspended within the water column, are a crucial component of stream ecosystems. Despite their importance, aquatic microbial communities are often treated as a black box in aquatic environments (Allison and Martiny, 2008). As land and water use alter biogeochemical fluxes and hydrological characteristics of aquatic ecosystems (Abbott et al., 2018; Blaszczak et al., 2019), understanding the drivers of microbial community composition is critical to assessing the scope of human impacts on freshwater systems (Lindström and Östman, 2011). Stream microbial communities are shaped by two interacting processes: environmental selection (Zwart et al., 2002; Fierer et al., 2007; Fierer and Lennon, 2011), and dispersal (Crump et al., 2007, 2012; Findlay, 2010; Savio et al., 2015; Albright and Martiny, 2018). Environmental selection leads to species sorting along stream habitats, with distinct microbial communities and interactions emerging across environmental gradients and habitat patches (Leibold et al., 2004; Niño-García et al., 2016). Dispersal, the movement of organisms from one environment or location into another, is particularly relevant for bacterioplankton, because these organisms are passively transported downstream with the constant movement of water.

**FIGURE 1:**
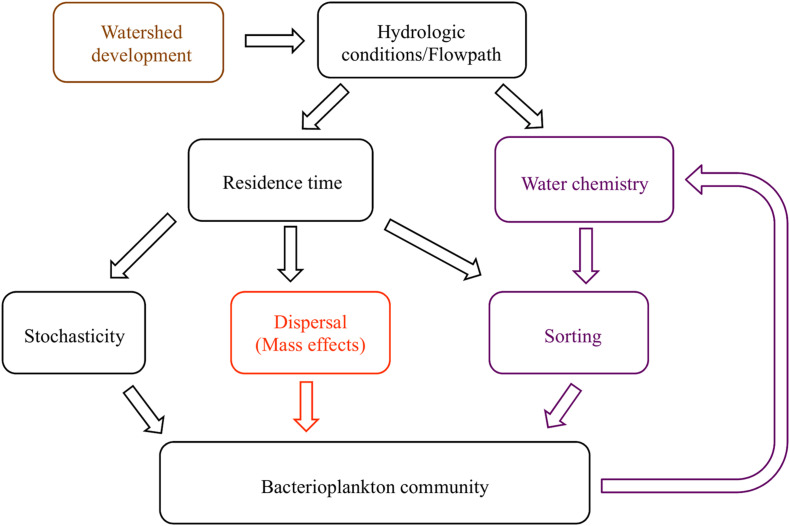
Conceptual diagram of the proposed relationships between hydrologic conditions and bacterioplankton communities. Residence time, a hydrologic condition related to flow velocity and volume, influences the extent that dispersal and stochastic processes alter community composition. Residence time also controls the extent to which environmental pressures act on communities to create species sorting, or selection. When dispersal is high, mass effects dominate; otherwise, dispersal limitation may occur. Bacterial metabolic activity affects water chemistry, creating a feedback loop within the model (hypothesis 1, purple). The community composition similarity along and between watersheds is related to longitudinal and lateral dispersal (hypothesis 2, orange). Anthropogenic changes to watersheds affect hydrologic conditions, and with repercussions on all aspects of the system (hypothesis 3, brown).

The most important environmental factors for structuring bacterial communities are pH, salinity, temperature, and dissolved oxygen, because of their role in controlling, or being controlled by, cellular activity (Fierer and Jackson, 2006; Fierer et al., 2007; Doherty et al., 2017). Water residence time moderates the duration that bacterial communities are influenced by the environment (Ben Maamar et al., 2015; Abbott et al., 2016; Niño-García et al., 2016), potentially leading to feedback loops where bacteria engineer new conditions that select for an altered set of taxa (Figure 1). For example, longer residence times lead to anoxic conditions at and below the sediment-water interface as microbial decomposition exceeds reaeration rates (Baker et al., 2000; Zarnetske et al., 2011). Once anoxic conditions are created, alternative terminal electron acceptor pathways become activated, with many subsequent changes in water chemistry; for example, anoxic ecosystems switch from net bacterial nitrification to denitrification (Briggs et al., 2013; Oldham et al., 2013; Kolbe et al., 2019). However, dozens of environmental parameters, including physicochemical conditions and solute concentrations, affect the abundance of bacterial taxa due to selective pressure (i.e., species sorting). Trace elements, such as molybdenum (a cofactor in the enzyme nitrogenase) and rare earth elements like lanthanum, may stimulate growth at low concentrations, but may also be toxic at high concentrations (Herrmann et al., 2016; Smedley and Kinniburgh, 2017). Macronutrients and ions generally considered limiting for autotrophic organisms, such as nitrogen (N), phosphorus (P), and potassium (K), may be less important for explaining bacterial community composition, because bacterial metabolic pathways are able to metabolize even recalcitrant substrates into more bioavailable species (Zeglin, 2015). Organic matter quality and quantity in soils and streams also correlate with specific bacterial communities and ultimately bacterial activity across a broad range of climates and bedrock materials (Gabor et al., 2014; Ruiz-González et al., 2015; Miller et al., 2016). Explaining bacterioplankton community taxonomic composition with instantaneous stream chemistry conditions is complicated by temporal changes in the source and flow path of water entering a channel (Leff and Lemke, 1998; Dahlke et al., 2012; Moatar et al., 2017). Seasonal changes in hydrology and environmental parameters affect the concentration, form, and downstream availability of organic and inorganic matter necessary for, or inhibitory to, microbial function (Duff and Triska, 2000; Hendricks and White, 2000).

The effect of selective pressure by the environment on a community is constrained by which taxa are present through dispersal. Stream-lake networks are unique because of their dendritic nature, which creates specific patterns in biodiversity (Widder et al., 2014). Bacterioplankton dispersal in streams is constrained to directional flow paths and, like stream water chemistry, is ultimately the combination of longitudinal (i.e., upstream to downstream in channel), vertical (groundwater-surface-water exchange), and lateral (i.e., stream-bank exchange and tributaries) contributions (Covino, 2017). Headwater inputs and longitudinal connectivity are crucial for maintaining downstream community composition and potentially biogeochemical function; alpha and beta diversity are highest in low-order streams, and decrease as stream order increases (Crump et al., 2007; Besemer et al., 2013; Savio et al., 2015). River systems are naturally punctuated by lakes and other abrupt changes in the natural topography (Ward and Stanford, 1983). The role of artificial reservoirs in driving bacterioplankton communities has become increasingly important as they store an estimated four times the volume river water (2,000 km^3^, compared to 8,000 km^3^) with an additional 200,000 km^3^ in lakes (Shiklomanov, 1993; Messager et al., 2016; Abbott et al., 2019). Natural streams are less of a gradual longitudinal continuum and more often a series of highly distinct environments, particularly when considering microhabitats of bacteria, but lakes and reservoirs have been incorporated into a limited number of studies of riverine bacterial communities, including large rivers (Ruiz-González et al., 2013; Chen et al., 2020), and arctic and boreal lake networks (Adams et al., 2014; Niño-García et al., 2016).

Discontinuity in modern streams comes from both natural and human sources as humans have increasingly altered landscapes and introduced infrastructure along rivers that impede the flow of water and associated matter (Grimm et al., 2008; Hale et al., 2015; Grill et al., 2019). Links among bacterial communities, stream characteristics, and dispersal must consider the influence that urbanization exerts on natural processes due to increased infrastructure development to support growing populations in nearly every aspect of streams and rivers (Meybeck, 2003). Reservoirs and regulated lakes (natural lakes modified with infrastructure to provide managers with control over flow and lake elevation) behave differently than natural lakes (Döll et al., 2009), so understanding their unique impact on stream bacterial communities is crucial. Land use changes, such as agriculture, mining, forestry, and urbanization, may result in less direct changes to stream conditions by impacting hydrologic connections between upland, groundwater, and stream ecosystems (Rose and Peters, 2001; Meybeck, 2003). An estimated 77% of global land area is affected directly by land use change; indirect anthropogenic effects increase that number to 100% (Vitousek et al., 1997; Watson et al., 2018). Disturbance of the land surface affects water flowpath and chemistry, altering the microbial community in soils, aquifers, and surface waters (Covino, 2017; Moatar et al., 2017). Without more comprehensive understanding of the controls on stream bacterioplankton, it is almost impossible to account for the complex interactions between land use change, altered hydrology, and water chemistry, and alterations in bacterial communities (Fisher et al., 2015; Van Rossum et al., 2015; Hall et al., 2018).

Northern Utah streams provide an ideal setting to understand anthropogenic and seasonal impacts on bacterioplankton and the interactions between water chemistry and bacterial communities. Land uses in the region include agriculture, forestry, mining and urban development, with built infrastructure encompassing reservoirs, irrigation systems, cross-basin diversions and flood control to support human industries (Hall et al., 2015). To understand the effect of environmental conditions, dispersal mechanisms, and human infrastructure on stream bacterioplankton community composition, we collected bacterioplankton and a suite of environmental parameters from five locations along montane to urban gradients in three northern Utah watersheds, over three seasons (fall, spring, and winter). We hypothesized that: (1) bacterioplankton communities will correlate with standard water quality metrics, (pH, temperature, specific conductivity, and dissolved oxygen), more than other groups of variables (nutrients, major ions, trace elements and organic matter) because of their importance in regulating essential metabolic processes, unless basic physicochemical conditions are relatively homogeneous (Lindström et al., 2005; Fierer and Jackson, 2006; Niño-García et al., 2016; Henson et al., 2017); (2) Bacterial communities will be less diverse below reservoirs, possibly due to dispersal limitation or species sorting within reservoirs, corresponding to increased residence time (Lindström and Östman, 2011), and (3) the bacterioplankton communities in urban environments will be less diverse than sub alpine streams, possibly due to the homogenization of hydrologic conditions and flowpath (Figure 1).

## Materials and Methods

### Study Sites

**FIGURE 2:**
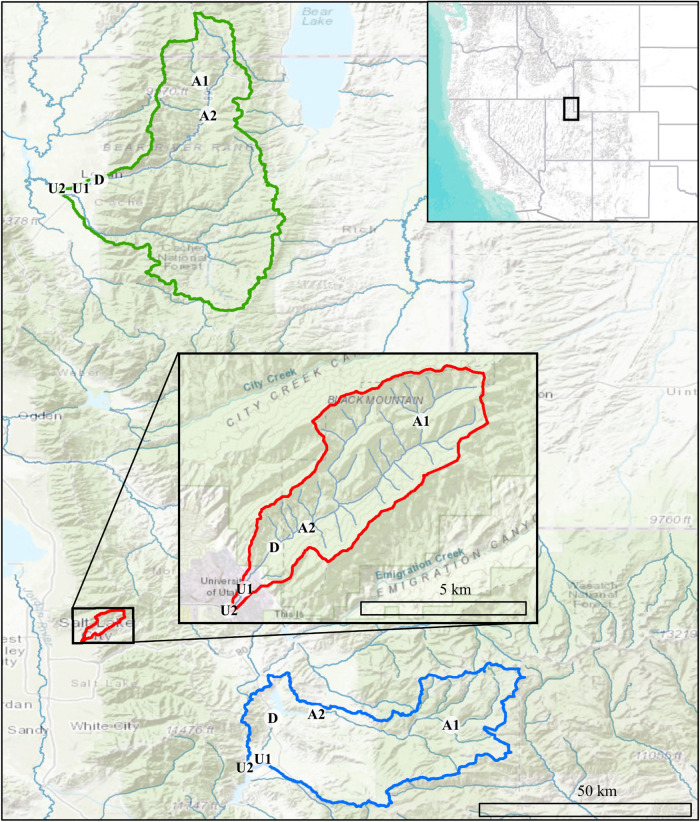
Map showing Logan (green), Red Butte (red), and Provo (blue) Watersheds in the Wasatch Range Metropolitan Area and locations within watersheds where bacterial communities and water quality data were collected. Location markers indicate position relative to man-made reservoirs and urban centers in all three watersheds: A, Above; D, below Dam; U, Urban. Site metadata is included in Table 1. Sources: ESRI, USGS, NOAA.

Our project was conducted in three northern Utah, USA watersheds, selected as part of the iUTAH (innovative Urban Transitions and Aridregion Hydro-sustainability) project, funded by the NSF Established Program to Stimulate Competitive Research (EPSCoR) (Jones et al., 2017). The watersheds include Red Butte Creek, Logan River, and Provo River (Figure 2). Within these watersheds, long-term stream sampling sites were selected from subalpine (up to 2,368 m.a.s.l) elevations to low-elevation (down to 1,353 m.a.s.l.) urban and agricultural land uses to capture the effects of elevation and urbanization on water resources (Table 1). Snowmelt-dependent streams in the region flow from undeveloped mountains into densely populated valleys. In the three study watersheds, this transition is demarcated by dams of varying sizes built to meet urban and agricultural water demand in the semi-arid climate of the basins below. Logan River passes through a series of smaller impoundments compared to the other watersheds, with shallow reservoirs and much lower water residence times (Table 2). We included sites above and immediately downstream of the reservoirs to measure the effect of the introduced infrastructure on the bacterial community composition.

**TABLE 1:** Stream site characteristics in the Logan, Red Butte, and Provo River Watersheds (Figure 2).

Watershed	Location	Site name	Elevation (m.a.s.l.)	Distance from outlet (km)	Watershed area (km^2^)	% Developed	Discharge (m^3^ s^–1^)
Logan	Above1	Franklin Basin	2110	75.67	63.4	0.0	1.05
Logan	Above2	Tony Grove	1886	62.86	277.6	0.1	1.75
Logan	Dam	Water Lab	1414	17.34	556.4	0.2	4.14
Logan	Urban1	Main Street	1377	11.49	560.3	0.6	3.14
Logan	Urban2	Mendon Road	1353	0	1924.6	0.9	4.64
Red Butte	Above1	Knowlton Fork	1986	9.00	3.7	0.0	0.020
Red Butte	Above2	Above RB Reservoir	1649	3.72	18.7	0.0	0.047
Red Butte	Dam	Red Butte Gate	1582	2.45	20.6	0.0	0.042
Red Butte	Urban1	Cottam’s Grove	1502	0.85	22.4	0.3	0.038
Red Butte	Urban2	Foothill Drive	1449	0	22.8	1.3	0.032
Provo	Above1	Soapstone	2368	75.73	154.7	0.2	3.25
Provo	Above2	Hailstone	1880	31.07	589.6	0.2	7.25
Provo	Dam	Below Jordanelle	1790	20.42	672.5	0.2	7.31
Provo	Urban1	Lower Midway	1676	4.65	718.5	0.3	5.44
Provo	Urban2	Charleston	1658	0	779.6	1.3	6.27

*Location indicates site position relative to reservoir(s) in the watershed, with Dam referring to sites immediately downstream of reservoir outflow (Table 2). Percent developed was calculated for the entire upstream watershed using NLCD 2011 data, not including “developed open space” (green space within urban areas). Mean discharge (and standard deviation) was calculated based on the 2014 water year, which is when sampling occurred.*

**TABLE 2:** Physical specifications and discharge of the reservoirs and dams within the three study reaches.

Watershed	Logan	Red Butte	Provo
Reservoir	First Dam	Red Butte	Jordanelle
Volume at capacity (m^3^)	172687	474890	395083644
Residence time (day)	0.5	43	1067
Dam height (meter)	9.14	39	105.16
Average discharge (m^3^ s^–1^)	4.14	0.042	7.32
Average discharge November 2014 (Fall)	2.87	0.02	4.21
Average discharge February 2015 (Winter)	2.92	0.05	4.24
Average discharge May 2015 (Spring)	10.01	0.13	8.59

*The Logan Watershed has a series of multiple impoundments with the lowest, First Dam, being the largest. Using maximum capacity and discharge at gages located immediately downstream, we calculated annual residence time.*

The degree and type of watershed development is variable within and among the three watersheds. The headwaters of each watershed are federal land and have some degree of protection from urbanization. Red Butte Creek has the most stringent protections; its headwaters are designated as a natural research preserve. The most impacted headwaters are in the Logan River watershed, which is opened to livestock grazing each summer (Hall et al., 2015). Each of the three watersheds’ valleys has a unique urban development type, allowing a comparison of a range of human activity. The Red Butte Creek watershed experienced urbanization beginning two centuries ago with Fort Douglas, one of the oldest permanently maintained developments in Utah; the subsequent construction of the University of Utah campus and residential areas exclude any present-day agricultural land use (Ehleringer et al., 1992). The Logan River watershed has valley sites that are a mixture of agriculture with some urban land uses, as the slow population growth in Cache County has resulted in a gradual shift from an agriculture-dominant to urbanized landscape. The middle region of the Provo River is also shifting from agricultural land use to newer urban development and was designated the fastest growing area in the country in 2016 (4.7% annual increase in population, US Census Bureau, 2017).

### Bacterioplankton Community Composition

We designed a sampling regime to capture longitudinal urbanization gradients and the effect of seasonal changes in hydrology and environmental conditions on bacterioplankton communities. We collected water column samples at fifteen sites, including five locations (named for position relative to reservoirs) in each of the three study watersheds (Figure 2). Streams were sampled in November 2014 (Fall), which is dominated by low-flow conditions and subsequently high residence times, with leaf litter potentially providing organic matter subsidies to inorganic groundwater contributions; in February 2015 (Winter) to capture low-flow, snow-covered conditions when flows and inputs are likely most homogenous within and across watersheds; and in May 2015 (Spring) to capture peak runoff conditions, when residence times are lowest and lateral connectivity is high. We chose to collect suspended bacteria because these communities are highly responsive to urbanization- and season-driven changes in water residence time, and sampling them can capture spatial and temporal shifts in bacterial dispersal influenced by the surrounding watershed along the stream continuum. Focusing on bacterioplankton also allowed us to collect a more comprehensive sample and eliminate possible differences due to cross-site variable streambed material, which influences biofilm establishment based on size fraction and mineral type (Donlan, 2002; McCormick et al., 2014).

We used a metabarcoding approach for identifying bacterioplankton community composition. In the field, we filtered water onto 47-mm 0.2-μm PES Supor filters (Pall) using autoclaved filter cups (Nalgene) until filters were clogged, which varied between 100 and 700 mL (adapted from Somerville et al., 1989). We stored filters in cryovials in liquid nitrogen to immediately suspend microbial activity. We stored samples at −80°C until extracting with PowerSoil DNA extraction kits according to manufacturer instructions (MOBIO). We PCR-amplified the V4 region of the bacterial 16S rRNA gene with primer set 515F and 806R (Caporaso et al., 2011). After checking that amplification proceeded normally using gel electrophoresis, we purified and normalized samples (SequalPrep Normalization Plate Kit, Invitrogen). Samples were submitted to the Brigham Young University DNA Sequencing Center^1^ for 2 × 250 base pair paired-end sequencing on an Illumina HiSeq 2500 System. We processed sequences using a modified Mothur pipeline using SILVA-based reference alignment version 128 (Schloss et al., 2009; Quast et al., 2013). We calculated the relative abundances of bacterial taxa at the 97% operational taxonomic unit (OTU) similarity cutoff. All community inferences were based on 41 samples with 138,458 total sequences rarefied to 15,445 sequences, and 1,450 unique OTUs with samples possessing an average sequencing coverage of 90.1% ± 0.62 (mean and standard error). Four samples were removed during QA/QC due to low read counts including: Provo Above2 Fall, Logan Above1 Winter, Logan Dam Winter, and Logan Below1 Winter. All bacterial sequences are available at http://www.hydroshare.org/resource/48fc6871c51d436b83000a8d29ddb702, and code used in Mothur can be downloaded at https://github.com/erinfjones.

### Environmental Factors

We quantified a wide range of environmental variables concurrent with bacterial sampling to identify which parameters correlated to community changes (see section “Environmental Drivers of Bacterioplankton Communities”). We measured standard water quality parameters (pH, water temperature, dissolved oxygen, conductivity) using either a YSI Quatro multiparameter probe or YSI EXO2 sonde (data downloaded from iUTAH web services using the R package WaterML; Kadlec et al., 2015; Jones et al., 2017). We analyzed nutrients potentially related to bacterial activity, including total nitrogen (TN, persulfate oxidation digestion and cadmium reduction method), total phosphorus (TP, persulfate oxidation digestion and ascorbic acid method), nitrate (EPA 353.2), ammonia (EPA 350.1), and dissolved orthophosphate (EPA 365.1) colorimetrically on an autoanalyzer (Astoria-Pacific). Total and volatile suspended solids were determined from combustion of pre-ashed Glass Fiber Filters (GF/F, Whatman) at 450°C for 2 h, and chlorophyll α was analyzed using ethanol extraction of filters followed by analysis on a handheld Turner Aquaflor fluorometer (Sartory and Grobbelaar, 1984; Steinman et al., 2017). Dissolved organic carbon (DOC), determined by acidification or sparging of inorganic carbon followed by combustion catalytic oxidation and NDIR detection, and total dissolved nitrogen (TDN), using the catalytic thermal decomposition/chemiluminescence method, were determined using the Shimadzu TOC analyzer. We measured major anion concentrations (F, Cl, and SO_4_) on a Dionex ICS-90 ion chromatograph. Major cations (Ca, K, Mg, Na) and trace elements (Ag, Al, As, B, Ba, Be, Cd, Ce, Co, Cr, Cs, Cu, Eu, Fe, La, Li, Lu, Mn, Mo, Nd, Ni, Pb, Rb, Sb, Se, Sm, Sr, Tb, Ti, Tl, U, V, Y, and Zn), which potentially shape bacterial community structure (Zeglin, 2015), were measured using an Agilent 7500ce quadrupole inductively coupled plasma mass spectrometer (ICP-MS, Goodsell et al., 2017). Stable isotopes in water (δ^18^O and δD), which we included with trace elements as a surrogate of water source (Follstad Shah et al., 2019), were measured on unfiltered aliquots using a Los Gatos Research Liquid Water Isotope Analyzer (LWIA-24d, Carling et al., 2015).

We characterized spectrofluorometric properties of dissolved organic matter (hereafter “organic matter”) from excitation emission matrices (EEMs) using a Horiba Aqualog spectrofluorometer (Horiba Scientific). EEMs were collected over excitation wavelengths 248–830 nm at 6 nm increments and over emissions 249.4–827.7 nm at 4.7 nm (8 pixel) increments. All samples were collected in Signal/Reference ratio mode to control for fluctuations in light source, and samples that exceeded 0.3 absorbance units at excitation 254 nm were diluted with deionized water. All samples were corrected for inner filter effects, Rayleigh scatter, and blank subtracted in MATLAB^TM^ (version 6.9; MathWorks) as described in Murphy et al. (2013). We calculated six indices from the EEMs, including: the beta:alpha index (BIX), where higher values represent more microbially derived DOM (Parlanti et al., 2000; Huguet et al., 2009); humification index (HIX) with higher values representing more humic-like material (Zsolnay et al., 1999); fluorescence index (FI), a ratio of fulvic- vs. humic-like organic matter (McKnight et al., 2001); TC index, the ratio of maximum fluorescence in the peak T region (protein-like) versus peak C region (humic-like), with higher values representing more protein-like organic matter, including WWTP effluent (Baker et al., 2008); SUVA_254_, specific ultraviolet absorbance at 254 nm, an indicator of aromaticity (Weishaar et al., 2003); and Total EEM intensity, which correlates to the concentration of organic carbon in the sample.

EEMS were also used to resolve a 4 component PARAFAC model following protocols outlined in Supplementary Methods (Murphy et al., 2013). Components characterized as humic-like or protein like based on peak location and comparison to previously reported fluorophores in the OpenFluor library (Murphy et al., 2014). Component 1 represented percent organic matter within component 1 (humic-like, developed), component 2 (humic-like, forested), component 3 (protein-like, tryptophan-like, developed), and component 4 (protein-like, tyrosine and tryptophan, forested); and % Protein, the percent protein-like organic matter as indicated by the sum of components 3 and 4 (Supplementary Figure 1).

We also calculated two land use parameters that may explain changes in bacterioplankton communities: percent developed area (not including developed open space such as parks) and percent impervious surface, using 2011 National Land Cover Database (NLCD) and watersheds delineated from 10-m digital elevation models (DEM) from the Utah Automated Geographic Reference Center (AGRC) in ESRI ArcMap 10 (Van Rossum et al., 2015; Chen et al., 2018).

### Statistical Analysis

#### Environmental Drivers of Bacterioplankton Communities

To evaluate the effect of environmental factors on the bacterial communities, we grouped variables into five categories and generated redundancy analyses (RDA) for each category. The five categories were standard field parameters (watershed, season, elevation,% impervious surface, pH, DO, temperature, specific conductivity, turbidity), nutrients (NH_4_, NO_3_, TDN, PO_4_, TN, DOC, SO_4_), major ions (Na, Mg, K, Ca, F, Cl), trace elements and isotopes (Li, B, Al, V, Mn, Fe, Co, Ni, Cu, Zn, As, Se, Rb, Sr, Y, Mo, Sb, Ba, La, Ce, Eu, Pb, U, δ18O, δD), and dissolved organic matter (BIX, HIX, FI, TC, SUVA_254_, Total intensity, % Protein, component 1, component 2, component 3, component 4). For each model, we calculated Pearsons correlation values and removed variables with multiple exceedances of *R* > 0.8 one at a time until all values were below < 0.8 to reduce autocorrelation. We selected marginally significant variables for each model (*P* < 0.1) using backwards step-wise regression using the R package vegan (Zelenı, 2011; Oksanen et al., 2015). We used RDA and not canonical correspondence analysis (CCA) after determining that our environmental variables followed a linear and not unimodal distribution (DCA axis lengths < 3.0, Lepš and Šmilauer, 2003). We calculated variance inflation factor (VIF), and removed any variables with VIF scores greater than 6 (James et al., 2013). For each RDA, we report adjusted R^2^ (the percent of variability in community composition explained, calculated by a PERMANOVA test of the model), constrained proportion (cp; the amount of variability in community composition explained with environmental variables, i.e., the constrained model, compared to without, i.e., the unconstrained model), and axis values (the percent of community composition explained by the first and second ordination axes). Ordination plots of models 1–5 are included in Supplementary Figures 2–6. Variables selected by RDA models 1–4 were combined and used in a sixth backward step-wise regression. Model 5, correlating dissolved organic matter characterization with communities, was generated using a subset of only 19 samples for which EEMs were available; to avoid reducing the number of samples included in the combined model or skewing results by attempting to interpolate missing values, we excluded organic matter variables in the combined model. Model 7 includes the sampling design factors. We also used variation partitioning to test the variability in community correlated to each matrix of environmental factors against principal coordinates of neighbor matrices (PCNM), which we calculated from distance matrices of latitude and longitude data (Buttigieg and Ramette, 2014). The variables selected by the combined model were rescaled and tested for differences between sites, watersheds, and seasons using ANOVA and TukeyHSD, histograms of residuals showed statistical assumptions were met.

#### Bacterioplankton Community Dynamics Along the Stream Continuum

Community similarity between watersheds, locations, and seasons was calculated using principal coordinate analysis (PCoA) of Bray-Curtis dissimilarities (phyloseq package; McMurdie and Holmes, 2013) and statistically tested using permutational multivariate analysis of variance (PERMANOVA) and visualized using ggplot2 (Wickham, 2016). To determine which of the watersheds, locations or seasons were different from the others, we used pairwise PERMANOVA tests, using the Holm method to correct for multiple comparisons (pairwise.Adonis R package; Martinez Arbizu, 2019). We tested for changes in bacterial diversity related to watershed, location, and season by calculating observed richness and Shannon diversity index. Values were graphed using box and whisker plots in R package ggplot2, with the box indicating interquartile range and whiskers showing the high and low extent of observations, with medians shown as a middle bar (Wickham, 2016).

#### Human Influence on Bacterioplankton Communities

We built community co-occurrence network models to compare the core community topology by analyzing samples grouped by watershed. The models were based on the maximal information coefficient (MIC) analysis in R package minerva (Reshef et al., 2011; Filosi et al., 2014). The nodes in the network models represent OTUs and edges represent significant co-occurrence connections that occur in at least 75% of samples in each watershed and have an MIC that is both > 0.7 and statistically significant (*P* < 0.01; Junker and Schreiber, 2011). We exported the graphs from R using igraph into Gephi (v. 0.8.2-beta; Bastian et al., 2009), where we visualized networks and calculated network statistics (Campbell, 2015).

To identify which taxa had unique patterns in abundance, we used analysis of composition of microbiomes (ANCOM) to determine which taxa (grouped by family) were significantly different in relative abundance between watersheds when controlled for location and season (Mandal et al., 2015). The results of the ANCOM were visualized using heatmap (R basic), where darker color indicates higher relative abundance. Samples and families are clustered by similarity in relative abundance, indicated by dendrograms on relevant axes (i.e., trees do not represent phylogeny of taxa). We calculated changes in percent relative abundance between different categories and reported means and standard deviations. All R code used for analysis is available at https://github.com/erinfjones/GAMUTdownload.

## Results

### Environmental Drivers of Bacterioplankton Communities

Of the six RDA models, encompassing 53 environmental variables, trace elements and isotopes explained the most community variation [*R*^2^ = 0.22, *P* = 0.001, *df* = 6, constrained proportion (cp) = 0.37; Table 3], while nutrients (*R*^2^ = 0.09, *P* = 0.001, *df* = 4, cp = 0.19) and major ions (*R*^2^ = 0.08, *P* = 0.001, *df* = 3, cp = 0.15) explained the least. Models incorporating standard field parameters (*R*^2^ = 0.15, *P* = 0.001, *df* = 5, cp = 0.25) and organic matter (*R*^2^ = 0.16, *P* = 0.001, *df* = 5, cp = 0.35) were intermediate in explaining bacterial community composition.

**TABLE 3:** Redundancy analysis (RDA) model results indicating the stream physiochemical variables structuring bacterial communities in streams across three watersheds (Logan, Red Butte, and Provo) and three seasons (Fall, Winter, and Spring).

#	Model name	Variables	Significant variables	Adj. *R*^2^	Axis 1, Axis 2 (%)	CP
1	Standard field parameters	Watershed, Season, Elevation,% Imp., Temp, pH, DO, Sp. Cond, Turbidity	Watershed, Temp, pH, Sp. Cond	0.147	39.46, 21.08	0.2534
2	Nutrients	NH_4_, NO_3_, TDN, PO_4_, TN, DOC, SO_4_	NO_3_, DOC, SO_4_, NH_4_	0.086	35.21, 30.18	0.1909
3	Major ions	Na, Mg, K, Ca, F, Cl	Mg, K, F	0.077	50.55, 30.41	0.1565
4	Trace elements	B, Mn, Co, Cu, Zn, As, Se, Rb, Ce, Eu, δ18O	B, Mn, As, Se, Ce, δ18O	0.216	33.23, 19.86	0.3726
5	Organic matter	BIX, HIX, FI, TC, TotInten, pFmax1, pFmax2, pFmax3, pFmax4, pProtein, SUVA	FI, TC, pFmax2, pFmax4	0.158	39.67, 29.44	0.345
6	Combined	Significant factors from models 1–4	Temp, NO_3_, F, B, Mn, Se, Ce, δ18O	0.251	27.12, 17.28	0.4508
7	Sampling design	Watershed + Season + Location	Watershed, Season, Location	0.183	33.7, 16.64	0.3464

*Environmental variables were separated into five categories and analyzed separately using backward stepwise selection of AIC scores to identify marginally significant components (p < 0.1). Variables from the best-fit models (excluding number five, which used fewer samples than other models) were combined and reported as an overall model. We report summary statistics, including adjusted R^2^, RDA axis percents, and proportion of community variation explained by constrained model (CP). Visualizations of the ordinations are included in Supplementary Figures 2–6. All models were significant (p = 0.001).*

**FIGURE 3:**
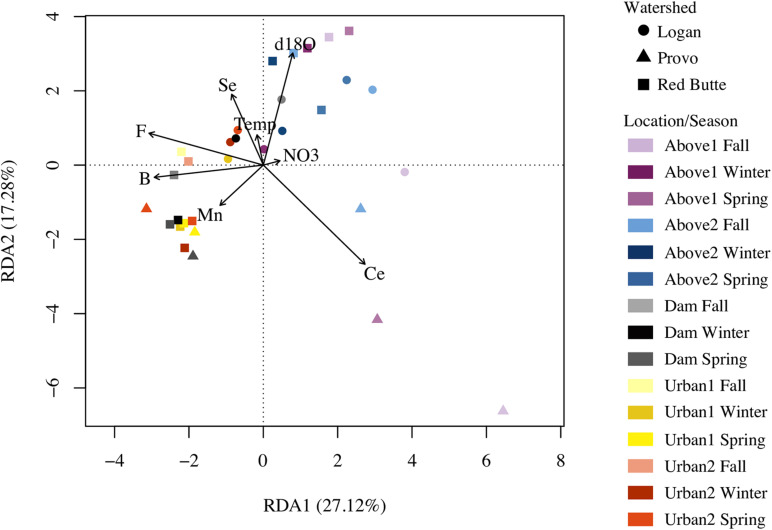
Redundancy analysis (RDA) plot relating stream environmental variables and bacterial communities in streams in three Utah watersheds (Red Butte Creek, Provo River, and Logan River) across three seasons (Fall, Winter, and Spring). Vectors are significant environmental factors, indicating positive correlation. Location indicates position relative to man-made reservoirs and urban centers. Variables were selected from the combination of four RDA models of standard field parameters, nutrients, major ions, and trace elements (Table 3).

The combined RDA model, incorporating significant variables from models one through four, included B, Ce, F, Mn, Se, NO_3_^–^-N, temperature, and δ18O, and explained more community variation than any other RDA (adj. *R*^2^ = 0.25, *P* = 0.001, *df* = 8, cp = 0.45; Table 3). Positive associations (represented by arrows indicating direction and magnitude of correlation between environmental factors and communities) occurred between high elevation Provo River bacterial taxa and Ce concentration, Red Butte Creek bacteria exiting the reservoir with Mn, and Logan stream bacteria, Se concentration, and temperature (Figure 3). Boron, F, and δ18O corresponded most with bacterial communities in Dam and Urban bacterioplankton communities in Red Butte and Provo watersheds. The parameters from the combined model explained 33% of the bacterial community variability (variation partition analysis, Supplementary Table 1). For comparison, the sampling design (Watershed + Location + Season) explained 43% of variation in the bacterial community.

Trace elements and the combined model correlated with the most amount of change in bacterioplankton community in the variation partitioning analysis (adj. *R*^2^ = 0.41 and 0.32; Table 4). Trace elements and the combined model had essentially no overlap in explanatory power between the environmental and spatial matrices (adj. *R*^2^ = −0.01). The overlap between variability explained by environmental and spatial factors was highest for standard field parameters, nutrients, and major ions (adj. *R*^2^ = 13–17%). Major ions explained no change in community composition that was not accounted for by the spatial matrix (adj. *R*^2^ = 0). Residual variability accounted for 48–80% of the community composition, indicating unaccounted for directional and stochastic processes.

**TABLE 4:** Variation partitioning of bacterioplankton community by environmental variables and spatial data shows trace elements explain more changes in composition than any other environmental or spatial factors.

#	Model name	Environmental (X1| X2)	Overlap	Spatial (X2| X1)	Residuals
1	Standard field parameters	0.23	0.13	0.05	0.59
2	Nutrients	0.03	0.17	0.01	0.80
3	Major ions	0.00	0.13	0.02	0.86
4	Trace elements	0.41	−0.01	0.11	0.48
5	Organic matter	0.21	0.07	0.13	0.59
6	Combined	0.32	−0.01	0.12	0.57

*Adjusted R^2^-values for the environmental matrix (X1) and spatial matrix (X2) excluding the other, the overlap between them, and the residual variability.*

**FIGURE 4:**
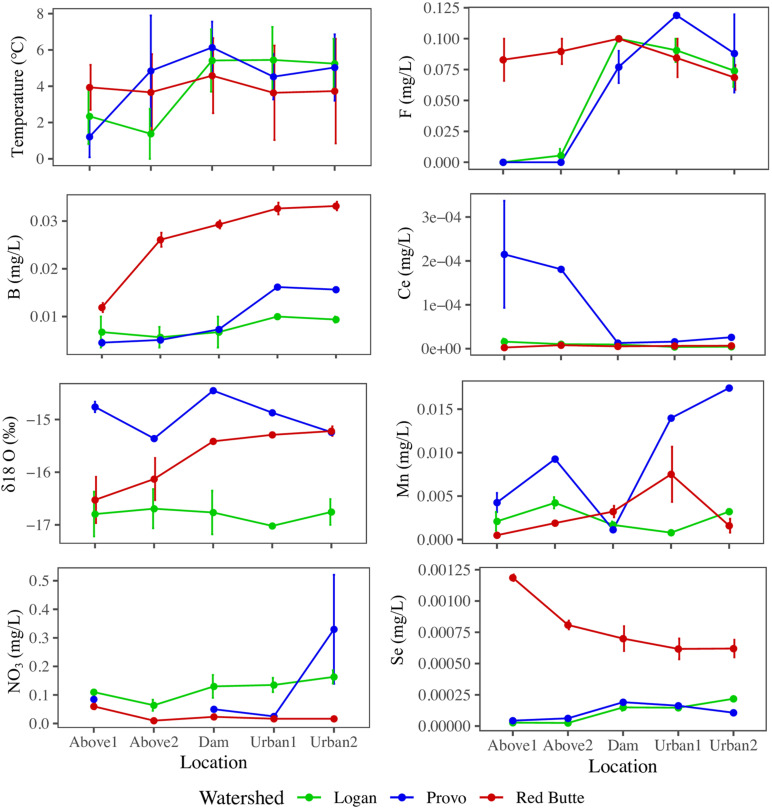
Longitudinal profiles of stream physiochemical variables selected by backward stepwise RDA ordination of bacterial communities in streams in three Utah watersheds (Red Butte Creek, Provo River, and Logan River) across three seasons (Fall, Winter, and Spring). Location indicates position relative to reservoirs and urban centers. Means and standard errors, where determinable, are shown (some data points were removed for QA/QC violations).

Variables used in the combined model (model 6) highlight the biogeochemical differences among watersheds and in urban environments (Figure 4). All parameters from the combined model except NO_3_^–^ and temperature differed by watershed, regardless of season and location (ANOVA, *P* < 0.05). Red Butte had the highest concentrations of B and Se. Some solutes increased between montane and urban sites (B, F), while only Ce decreased from upstream to downstream (TukeyHSD, *P-adj.* < 0.05). Boron, F, Ce, Mn, and temperature differed by location regardless of watershed (ANOVA, *P* < 0.05, *df* = 4) and also had significant changes due to the interaction of watershed and season (ANOVA, *P* < 0.05, *df* = 4). Cesium, Mn, and temperature were the only parameters in model 6 that varied with season alone (ANOVA, *P* < 0.05, *df* = 2).

### Bacterioplankton Community Dynamics Along the Stream Continuum

**FIGURE 5:**
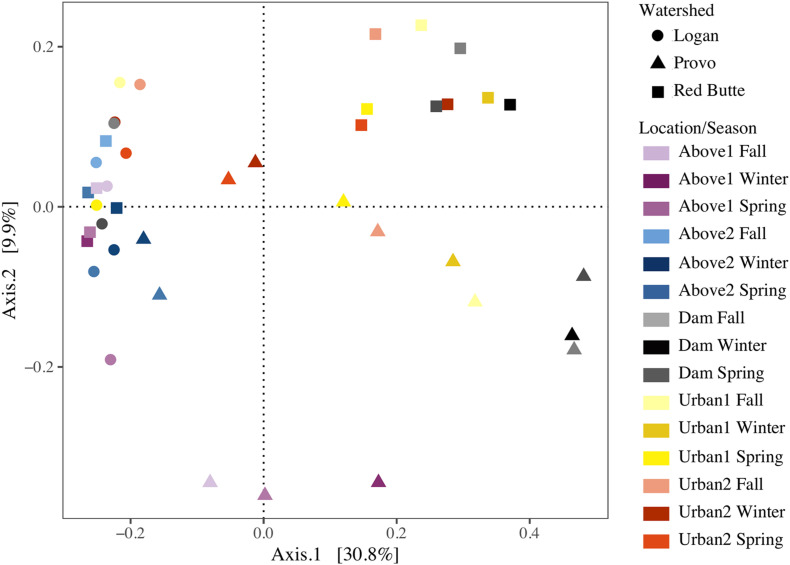
Reservoirs alter stream bacterial community composition from three watersheds in Utah’s Wasatch Range Metropolitan Area (Red Butte Creek, Provo River, and Logan River) across three seasons (Fall, Winter, and Spring). Graph represents a Principal Coordinate Analysis (PCOA) ordination, with each point representing a community of bacterioplankton classified at the 97% similarity from 16s rRNA gene amplicon sequencing of OTUs. Location indicates position relative to man-made reservoirs and urban centers. Points closer together are more similar (based on Bray-Curtis dissimilarity index), while points farther apart are dissimilar.

Bacterioplankton communities upstream of reservoirs were similar across all watersheds and seasons, despite large geographic distances and unique environmental conditions (Figure 5). Communities from the Logan watershed and upstream locations (Above1 and 2) in the Provo and Red Butte watersheds clustered together on the PCoA, indicating similar community compositions, but Dam sites in Provo and Red Butte watersheds, where dams increased water residence times by 2–4 orders of magnitude, were markedly unique. Downstream locations in the Red Butte watershed (Dam, Urban1 and 2) had a clear pattern of both location and season. Provo communities, where residence times in dams were longest, shifted further along Axis1 at Dam sites than Red Butte communities, then gradually moved back toward the Above and Logan watershed cluster downstream. Dam locations in Provo and Red Butte watersheds were most similar to the upstream community in May, when flows were highest and residence times lowest. The Logan watershed, despite covering the most stream miles (Table 1), was the most similar in community composition between sites.

The effect of watershed and location relative to season in driving bacterioplankton community composition was confirmed by PERMANOVA and pairwise *post hoc* PERMANOVA tests. The differences in community composition observed between groups in the PCoA ordination were significant for all three main effects (Watershed, Season, and Location), as well as the interactions of watershed with season and location (PERMANOVA, Supplementary Table 2, *P* = 0.001). The *R*^2^-values for watershed, location, and their interaction were each around 21%, while the R^2^ values for seasonal changes were much lower, around 8%. Logan watershed was the least similar to Provo and Red Butte (22 and 18%), according to pairwise PERMANOVA tests (Table 5). All watersheds were different from each other, regardless of season and/or location (i.e., *p*-values did not change when either were added as strata to the command model). The PERMANOVA test was unable to differentiate seasons, although controlling for location or watershed made the difference between Spring and Winter significant.

**TABLE 5:** Pairwise PERMANOVA results comparing bacterioplankton community composition across three watersheds, at five locations, and three seasons.

Watershed	Logan		Red Butte	Provo	
Logan	–		–	–	
Red Butte	0.18*		–	–	
Provo	0.22*		0.12*	–	

**Location**	**Above1**	**Above2**	**Dam**	**Urban1**	**Urban2**

Above1	–	–	–	–	–
Above2	0.09	–	–	–	–
Dam	0.22*	0.27*	–	–	–
Urban1	0.19*	0.21*	0.05	–	–
Urban2	0.16*	0.16*	0.11	0.05*	–

**Season**	**Fall**	**Winter**		**Spring**	

Fall	–	–		–	
Winter	0.05	–		–	
Spring	0.07	0.05		–	

Higher R^2^-values suggest more difference in community between two groups; smaller numbers are more similar in composition. *Indicates p-value < 0.05.

**FIGURE 6:**
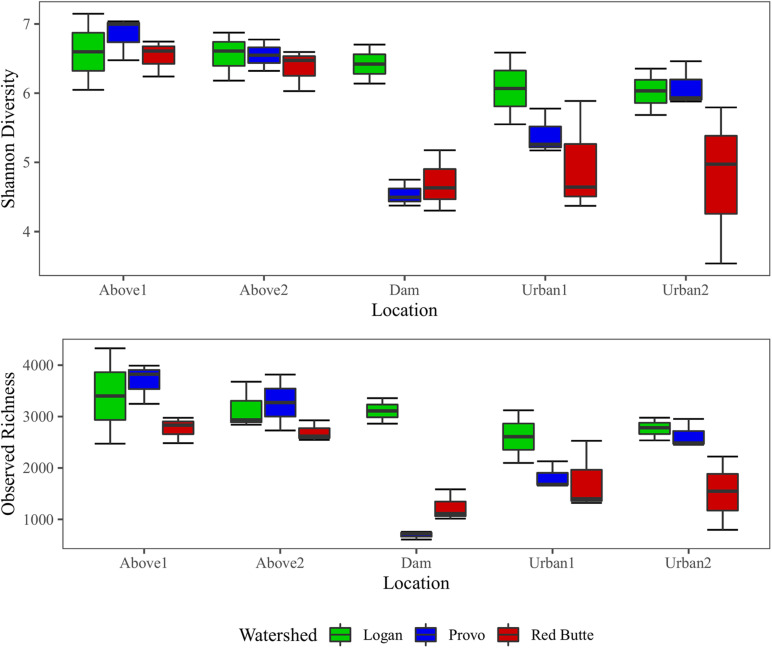
Observed richness and Shannon diversity of bacterial communities for three watersheds over three seasons in Wasatch Range Metropolitan Area (WRMA). Boxes depict interquartile ranges, with the center line on the median and whiskers showing the extent of values.

Both richness and diversity gradually decreased downstream in the Logan watershed, dropping by 18.7% (richness) and 8.7% (Shannon index) from upstream into the urban environment (Figure 6). Richness and diversity in Provo and Red Butte watersheds decreased slightly between Above1 and Above2 locations, then strikingly dropped at the Dam location. Diversity and richness then gradually increased downstream of the dam, despite moving into an urban environment, but remained slightly less diverse than the upstream communities. Richness and diversity were 25 and 11% higher for samples collected in Spring than Fall or Winter (TukeyHSD, adj. *P* < 0.05).

### Human Influence on Bacterioplankton Communities

Large reservoirs had substantial effects on bacterioplankton richness, diversity, and community composition. Both species richness and diversity were highest in the Logan watershed, where reservoirs are shallow with much lower residence times (TukeyHSD, adj. *P* < 0.05). In the other two watersheds, where reservoir residence time was higher, bacterial diversity decreased by 25% and taxa richness by 67% from upstream to downstream of large reservoirs. Location was selected as a component of the RDA model (Table 3), and in the pairwise PERMANOVA tests the Above2 and Dam locations had the most dissimilarity of any comparison in bacterioplankton communities between main effects (27%, Table 5).

Development on the landscape was comparatively less influential on bacterioplankton richness, diversity, and community composition. Richness and diversity increased downstream of dams in Provo and Red Butte watersheds, despite moving into an urban environment, but remained slightly less diverse than the headwater community (Figure 6). Percent developed land use was not included by the step-wise RDA model selection (Table 3). Despite being the most developed and in different ways (densely urban versus mostly agricultural), Red Butte and Provo watersheds were the most similar (Table 5). Dam, Urban1 and Urban2 locations were the most similar, and differences between them were not significant regardless of whether comparisons were corrected for watershed and season (data not shown).

**FIGURE 7:**
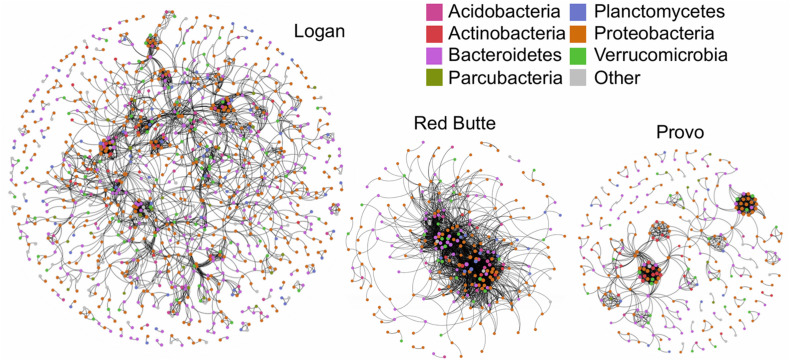
Network co-occurrence models for bacterial communities collected along elevation and urbanization gradients in three Utah watersheds (Red Butte Creek, Provo River, and Logan River) across three seasons (Fall, Winter, and Spring). Nodes indicate taxa (OTUs, taxa with 97% similar sequences) and edges connect where significant co-occurrence was detected in 75% of samples. Topologic statistics of the network are shown in Table 6.

The community network structure was unique for each of the three watersheds. Logan watershed communities had the highest co-occurrence network complexity, with 3–4 times as many nodes as the other two watersheds (Figure 7 and Table 6). Logan and Provo watersheds had similar modularities (the degree to which clustering occurs, 0.856 and 0.767), mean path lengths (the average number of steps to connect each node, 6.739 and 6.029), and mean degrees (average number of edges for each node, 5.533 and 6.662). Red Butte watershed had nearly as many edges as Logan, despite having much fewer nodes; as a result, Red Butte had a much more tightly clustered network, with over 5 times as high density (0.074), 3 times as high degree (19.83) and 0.5 times as high modularity (0.397) as the other two watersheds.

**TABLE 6:** Topological metrics calculated for network co-occurrence models of bacterial communities collected at five locations along an elevational gradient in three watersheds (Red Butte Creek, Provo River, and Logan River) across three seasons (Fall, Winter, and Spring).

	Logan	Red Butte	Provo
Nodes	1,071	270	305
Edges	2,963	2,677	1,016
Mean path length	6.739	3.452	6.029
Mean degree	5.533	19.83	6.662
Mean clustering coefficient	0.57	0.547	0.703
Density	0.005	0.074	0.022
Modularity	0.856	0.397	0.767

*Nodes indicate taxa (OTUs) and edges connect where significant co-occurrence was detected in 75% of samples, edges represent significant co-occurrence connections that occur in at least 75% of samples in each watershed and have an MIC that is both > 0.7 and statistically significant. Mean path length: the average number of steps to connect each node; Mean degree: average number of edges for each node; Mean clustering coefficient: a measure of how completely neighboring nodes are connected to each other.*

**FIGURE 8:**
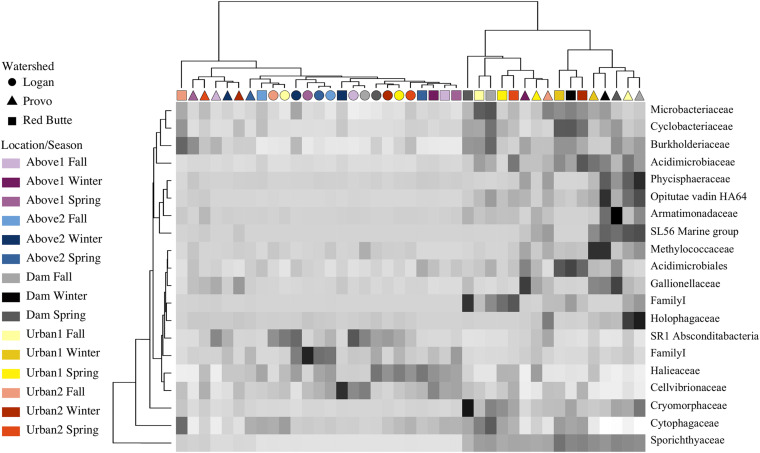
Heatmap showing ANCOM results of differentially abundant family for sites in three watersheds across three seasons (Fall, Winter, and Spring). Taxa were selected using a family relative abundance at least 0.5% for all samples. Location indicates position within watershed relative to man-made reservoirs, with Dam sites located immediately downstream of reservoir outlets (Table 1). Darker squares indicate higher relative abundance for bacterial families in each sample. Similarity between samples and taxa is indicated by dendrograms on each axis.

Relative bacterial taxa abundance had unique distribution patterns in each watershed, correlating with changes in residence time (large reservoirs) more than changes in catchment land use. The ANCOM test (displayed using an abundance heatmap in Figure 8) returned taxa unique across watershed, location, and season. Similarities of taxa grouped by family expression patterns (shown by dendrogram on *x*-axis) matched the clusters in the PCoA ordination. Sporichthyaceae (Actinobacteria) was an important component of communities at Dam and Urban sites in Red Butte and Provo watersheds (16.1 ± 6.9%), while absent from Logan watershed and Above locations (1.1 ± 3.1%). Cryomorphaceae (Bacteroidetes) were also enriched directly below reservoirs, with 3.8 ± 2.7% in Red Butte and Provo Dam sites, and only 0.3 ± 0.1% in Logan and Above sites. Red Butte watershed had higher relative abundance of Acidimicrobiales (Actinobacteria) in February (0.57 ± 0.07%), while Provo communities in February were enriched with methanotrophic Methylococcaceae (Gammaproteobacteria, 1.4 ± 0.01%). Logan watershed and Above locations had higher densities of Cellvibrionales (Gammaproteobacteria), including over six times the relative abundance of Halieaceae (0.88 ± 0.56%) and three times the relative abundance of Cellvibrionaceae (0.77 ± 0.59%). The most abundant taxa, the aerobic, motile Comamonadaceae (Betaproteobacteria; Willems, 2014), was present in all samples at relative abundances between 5.5 and 53.4% (mean of 13.3%), but was removed by the ANCOM test because it was not differentially abundant between watershed, season, or location.

## Discussion

### Environmental Drivers of Bacterioplankton Communities

Contrary to our hypothesis, trace element concentrations, and not standard field parameters like pH and dissolved oxygen, were best correlated with bacterioplankton communities. This finding is contrary to observations in soil bacterial communities and longitudinal riverine studies on the Amazon and Mississippi Rivers (Doherty et al., 2017; Henson et al., 2017). However, a recent meta-analysis of stream bacteria compositions found that trace metals, when included in ordinations, always correlated with community composition (Zeglin, 2015). Many studies that report the importance of standard chemical and physical parameters do not include trace elements in their analyses, possibly overestimating the bacterial variability correlated to the standard variables and failing to quantify the role of other unmeasured parameters.

Trace elements may capture the variability among bacterial communities due to unique variations in geology, groundwater, and resultant stream chemistry better than the relatively narrow range of conditions in pH (7.24–8.5), dissolved oxygen (8.4–14.7 mg O_2_/L), and temperature (0–9.5°C) in these streams. The correlation between bacterial community composition and δ18O was 19%, which indicates the importance of the source of water (age indicated by enrichment of δ18O either in groundwater or within reservoirs). Other solutes in the combined model (F, B, Mn, Se, and Ce) may indicate natural weathering of rocks within groundwater as well as urban pollution (Buszka et al., 2007), but either mechanism ties bacterioplankton community composition with the ultimate source of stream water.

Statistical testing of the 53 variables returned many differences between the watersheds, locations and seasons (data not shown), but in some cases these differences failed to impact bacterial community composition, while variables with no statistical difference did affect composition. For example, nitrate concentrations were statistically indistinguishable between any factor of watershed, season or location, but correlated to 10% of bacterioplankton community variation (variation partitioning analysis of significant RDA Model 2), demonstrating that ecological significance may occur where statistical significance does not. Trace elements are no longer included in many water quality studies because their concentration is usually in a narrow range below toxicity levels for aquatic organisms at higher trophic levels, but the slight differences in concentration might be high enough to differentiate the multitude of unique microbial taxa (Herrmann et al., 2016; Smedley and Kinniburgh, 2017).

Our highest variation partitioning adj. *R*^2^-value was 52% (model 4, sum of environmental and spatial factors), meaning we were unable to attribute half of community composition to any factor, which is similar to *R*^2^-values reported in a meta-analysis of 22 environmental microbiology studies (Hanson et al., 2012). Stochasticity, including genetic drift (e.g., die-off, bacterivory) and mutation (Hanson et al., 2012; Evans et al., 2017) may contribute to differences in bacterioplankton community proportional to residence time, but we were unable to distinguish their relative impacts with this study design. Our correlation values may underestimate the impact of environmental variables and overestimate dispersal and stochastic mechanisms compared to benthic biofilm communities (Wang et al., 2017). Bacterial dormancy and horizontal gene transfer (Trevors et al., 1987; Jones and Lennon, 2010), priority effects (Evans et al., 2017), competition between organisms, and trophic interactions such as predation (Pernthaler, 2005), may also account for part of the 50% of community composition we were unable to associate with environmental conditions.

### Bacterioplankton Community Dynamics Along the Stream Continuum

Our results appear to follow a pattern similar to other studies showing that the dominant dispersal pathway of stream bacterioplankton is via longitudinal transport from headwaters, where high lateral dispersal of bacteria from soil waters to streams helps form higher-diversity upstream communities that generally decrease in diversity downstream as environmental selection removes poorly adapted taxa (Crump et al., 2012; Besemer et al., 2013; Febria et al., 2015). Upstream of dams, diversity decreased with increasing stream distance as expected; however, the rapid decrease in diversity from above to below dams and then the gradual increase in diversity moving downstream demonstrated a deviation from typical patterns of bacterioplankton dispersal (Figure 6; Chen et al., 2018). The abrupt change in bacterioplankton community composition and diversity, presumably occurring within the reservoir, could be explained by a shift from dispersal-dominated community composition to a lower-diversity community shaped more by environmental selection. In reservoirs, longer residence times may separate bacterioplankton from both the longitudinal dispersal from upstream terrestrial soils (Lennon and Jones, 2011; Crump et al., 2012) and lateral dispersal from internal sources, such as lake sediments, that act as seed banks for microbial diversity (Comte et al., 2017). We observed a particularly atypical pattern along the stream continuum below reservoirs, with Provo and Red Butte community diversity increasing rather than decreasing downstream of dam sites. This downstream recovery in diversity could be caused by an increase in dispersal and decreased environmental selection as the bacterioplankton returned to a stream environment more influenced by dispersal and short residence times. Differences in stream-soil connectivity between these two watersheds may lend support to this idea; Red Butte Creek, which has a larger exposure ratio (surface area of bed:streamwater volume) than the Provo River, returned to the Above site PCoA cluster in much fewer stream kilometers than the Provo. Hydrologic variability, which is significantly decreased by static flows released from the reservoirs, may also play a role in structuring the bacterioplankton community indirectly through biofilm establishment (Widder et al., 2014).

Patterns in community similarity within and across watersheds could also support the idea that stream bacterioplankton communities are first dispersal-dominated and then increasingly influenced by environmental selection downstream. Most of the community similarity was attributed to watershed, possibly due to longitudinal transport of a common upstream-sourced community along the stream continuum. The lack of significant interaction between location and season (Supplementary Table 2) also indicates headwater-driven mass effects could play an important role in determining community composition, because an interaction would have indicated important contributions of unique local taxa based on changes in lateral sources and hydrologic connectivity through time. We expected to see a higher variability between communities due to seasonal changes in hydrologic conditions (Table 2), but a variation partitioning analysis of Model 7 determined only 7% of stream bacterial communities were associated with seasonal changes. Further, season was only significant in a pairwise PERMANOVA when controlled by watershed and/or location, suggesting that seasonal changes in residence time or lateral connectivity are less significant than expected (due to small and/or redundant contribution of bacterial taxa), unlike other stream bacterial communities that displayed strong seasonal changes (Leff et al., 1998; Yannarell et al., 2003; Crump and Hobbie, 2005; Zeglin, 2015; Niño-García et al., 2017).

### Human Influence on Bacterioplankton Communities

Developed land use, as indicated by percent impervious surface, was not associated with changes in bacterioplankton communities (Table 3, % Imp), unlike previous studies identifying correlations between the two (Van Rossum et al., 2015; Chen et al., 2018). However, bacterial community composition was strongly affected by reservoirs, as demonstrated by large changes in communities where reservoir residence time was high in both Red Butte and Provo watersheds (Table 2 and Figure 5). Moving into the urban environment, communities became more similar to the Above locations, suggesting that either (1) high lateral dispersal occurred from downstream soil communities, which were not strongly affected by development in these watersheds, or (2) changes in environmental conditions promoted taxa being transported from above the reservoir to once again increase in abundance. The community network topology had watershed-specific responses that may have been due to differences in residence time: Red Butte watershed maintained a consistent, highly connected core community while in Provo, taxa were less connected and may be comprised of more generalist taxa functioning independently (Figure 7). The network topology may be an artifact of the shorter stream distance and catchment area in the Red Butte watershed, which affected clustering of co-occurrence networks in an Austrian catchment (Widder et al., 2014). The reduced network may also suggest higher rates of hydrologic disturbance (Widder et al., 2014) or species sorting (Ren et al., 2019) and imply changes in community function between watersheds (Fuhrman, 2009). The negligible effect of changes in land use at higher order streams suggests instream bacterial communities may not always be dramatically affected by land use changes or stream channelization in urban areas.

Many environmental factors are affected by reservoirs that might be responsible for the change in bacterioplankton community below reservoirs (Lindström et al., 2005; Comte et al., 2017). Drastic changes in bacterial communities are reported in artificial water systems (e.g., stormwater outfall, sewage, and 9-km long drinking water delivery pipe), but these changes were mostly attributed to biofilm development within pipes (Fisher et al., 2015; Van Rossum et al., 2015). Another potential factor is the removal of the suspended sediment load and particle-associated bacteria. As water slows, sediment drops from the water column and removes a subset of the overall taxa that might be dispersing attached to soil particles in swift currents (Atkinson et al., 1992; Doherty et al., 2017). However, turbidity was not a significant factor in the RDA selection, suggesting that this mechanism was not responsible for the changes observed (Table 3).

Large reservoirs may also impact bacterioplankton communities by altering substrate availability (Ruiz-González et al., 2015). Carbon substrate quality changes depending on source, and large, deep lakes have very different carbon sources and cycles than shallow streams. As more labile carbon is metabolized in reservoirs, DOC levels increase. DOC concentrations correlated in an RDA ordination with the sites downstream of reservoirs (Supplementary Figure 2), suggesting that the carbon substrate availability was related to changes in the bacterial community (Barberán and Casamayor, 2011). BIX values, indicating an increase in autochthonous production, also correlated with sites below reservoirs (Supplementary Figure 5). Cryomorphaceae, which increased in relative abundance below reservoirs, are chemoorganotrophs that are thought to metabolize these simple organic compounds (Bowman, 2014a). Oxygen is another metabolic component that likely controlled changes in taxa with increased residence time in reservoirs. Unfortunately, because outflow from reservoirs was quickly re-aerated, the oxygen levels measured were not representative of the environment where the community was formed, and our models did not detect correlations between community composition and oxygen concentrations (Figure 3). For example, samples collected below the larger reservoirs had substantially higher relative abundance of the facultative anaerobe Sporichthyaceae (Tamura, 2014), suggesting the anoxic hypolimnion played a role in driving community change. Sulfur concentrations also correlated with below reservoir samples, possibly due to oxidation of hydrogen sulfide created by sulfur-reducing bacteria in reservoir sediments. Increased Methylococcaceae relative abundance in Provo reservoir outflows potentially indicates both methanogenesis and methanotrophy were also responsible for some of the changes in community composition (Bowman, 2014b). These changes in taxa may also correspond to an increase in mobilized heavy metals from reservoir sediments; for example, Acidimicrobiales in Red Butte outflow indicates iron-reduction and iron-oxidation may be increased within the reservoir (Clark and Norris, 1996; Itoh et al., 2011). Changes in bacterial taxa may relate to activation of alternative metabolic pathways, especially those rare metabolic processes for which functional redundancy may be low (Comte and del Giorgio, 2010; Peura et al., 2012; Adams et al., 2014).

## Conclusion

We tested three predictions regarding bacterioplankton community composition along a longitudinal-urbanization gradient, including multiple seasons and anthropogenic alterations to in-stream and landscape processes to determine the role of environmental conditions, water residence time, and human infrastructure on stream bacterioplankton community composition. Trace element concentrations explained more variability in bacterioplankton community composition than other environmental parameters, and should be included in more analyses of aquatic microbial ecology. Our findings followed previously established patterns showing that bacterioplankton diversity tends to gradually decrease downstream as environmental selection acts on dispersal-dominated communities, except in cases where large reservoirs drastically changed water residence time. Reservoirs and lakes (anywhere residence time changes) should be included in longitudinal stream bacteria studies; understanding changes in residence time may provide important context for other studies of bacterioplankton biogeography. Large reservoirs may have more impact on bacterioplankton communities than other aspects of watershed urbanization. More research is needed to quantify the magnitude of the effect of components of Figure 1, such as anthropogenic infrastructure and changes in residence time, on stream bacterial communities (e.g., when does longer residence time have increase or decrease alpha and beta diversity, and what controls the extent of that effect?). Understanding the drivers of bacteria and other microbial communities in streams will allow improved predictions of how watershed and stream development will affect biogeochemical processes and resultant water quality.

## Data Availability Statement

The datasets generated for this study can be found in the Hydroshare repository https://doi.org/10.4211/hs.c0a1bb39015444ef86ace38ae6c44e77.

## Author Contributions

EJ, ZA, and MB designed the study. EJ, NG, JK, and GC collected and analyzed samples. All authors helped write and review the manuscript.

## Conflict of Interest

The authors declare that the research was conducted in the absence of any commercial or financial relationships that could be construed as a potential conflict of interest.

## Footnotes

1 http://dnac.byu.edu/
