# Supplementary material for: Stream Microbial Community Structured by Trace Elements, Headwater Dispersal, and Large Reservoirs in Sub-Alpine and Urban Ecosystems
Source: Front Microbiol. 2020 Nov 26;11:491425. doi: 10.3389/fmicb.2020.491425 (PMC7726219; doi:10.3389/fmicb.2020.491425)
Supplement: Supplementary file 1 [file Data_Sheet_1.docx]

Supplementary Material

# Supplementary Methods

PARAFAC was used to identify humic and protein-like fluorescent components of DOM to elucidate differences in DOM that varied by watershed and location within a watershed. The drEEM toolbox was used to create a PARAFAC model in MATLAB^TM^ following Murphy et al. (2013). Resolved PARAFAC components were then compared to previously found fluorophores in the open source library OpenFluor (Murphy et al. 2014). A total of 499 EEMs, collected as part of a previous synoptic sampling effort from July 2014 to December 2015, were used to create the PARAFAC model. The model included EEMs from all 3 watersheds, each of which composed 11-36% of all EEMs used for the model. Of the EEMs from the model, 19 were collected concurrent with samples for this study, and were used for further analysis.

# Supplementary Tables

**Supplementary Table 1**. **Variation partitioning for stream physiochemical variables structuring bacterial communities in streams across three watersheds (Logan, Red Butte, and Provo) and three seasons (Fall, Winter, and Spring).** For each of seven ordinations comparing groups of environmental variables and bacterial community, we report variables and combinations of variables with the highest adjusted R^2^ values.

| **Variable** | **Model #** | **Adj. R2** |
| --- | --- | --- |
| Watershed | 1 | 0.1589 |
| Sp. Cond + Watershed | 1 | 0.2562 |
| NO3 + DOC + SO4 | 2 | 0.2013 |
| Mg + K | 3 | 0.1105 |
| δ18O | 4 | 0.1916 |
| B | 4 | 0.1442 |
| δ18O +NO3 + B + (Mn + Se + Temp + Ce + F) | 6 | 0.3312 |
| δ18O + B | 6 | 0.2706 |
| Watershed + Season + Location | 7 | 0.4362 |

**Supplementary Table 2.** **Analysis of variation (PERMANOVA) of** **bacterial communities in streams across three watersheds (Logan, Red Butte, and Provo) and three seasons (Fall, Winter, and Spring).**

Df SumsOfSqs MeanSqs F.Model R2 Pr(>F)

Watershed 2 1.8179 0.90894 10.6895 0.21505 0.001 ***

Location 4 1.7840 0.44600 5.2451 0.21104 0.001 ***

Season 2 0.5934 0.29668 3.4891 0.07019 0.001 ***

Watershed:Location 8 1.8570 0.23212 2.7299 0.21968 0.001 ***

Watershed:Season 4 0.6441 0.16103 1.8938 0.07620 0.007 **

Location:Season 8 0.7366 0.09207 1.0828 0.08713 0.340

Residuals 12 1.0204 0.08503 0.12071

Total 40 8.4533 1.00000

---

Signif. codes: 0 ‘***’ 0.001 ‘**’ 0.01 ‘*’ 0.05 ‘.’ 0.1 ‘ ’ 1

# Supplementary Figures


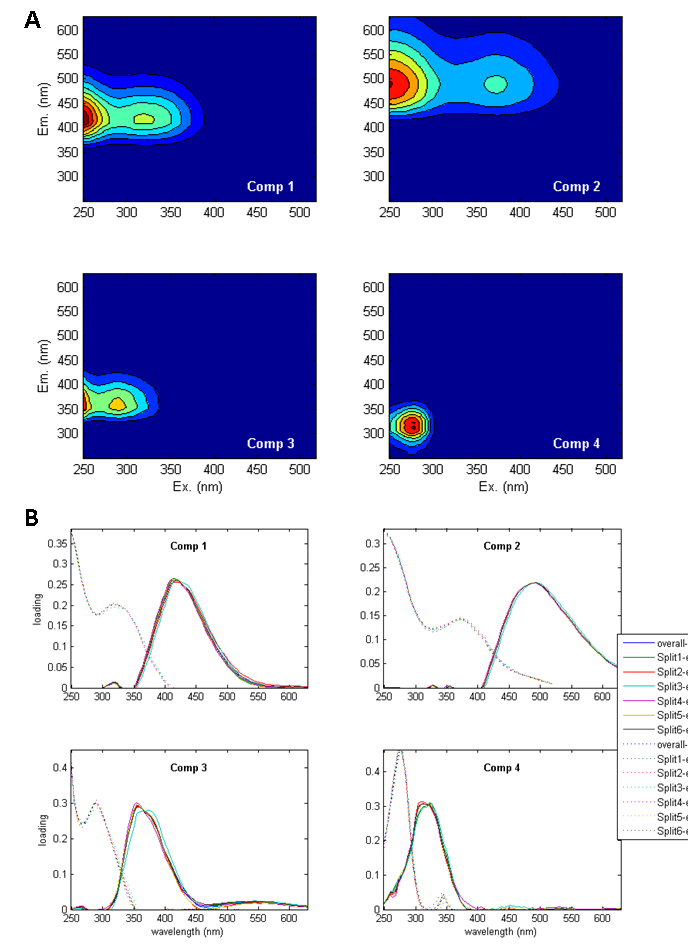


**Supplementary Figure 1.** A 4 component PARAFAC model was resolved (A) and validated with split-half analysis where split models found a match with Tucker correlation coefficient > .95 (B). Components 1 and 2 (C1, C2) were humic-like, and components 3 and 4 (C3, C4) were protein-like.

 **Supplementary Figure 2.** **Redundancy analysis (RDA) plot relating basic stream chemistry and physical characteristics with bacterial communities in streams in three Utah watersheds (Red Butte Creek, Provo River, and Logan River) across three seasons (Fall, Winter, and Spring).** Vectors represent positive correlations between environmental factors and a sample community composition. Location indicates position relative to man-made reservoirs and urban centers.

 ** Supplementary Figure 3.** **Redundancy analysis (RDA) plot relating stream nutrient chemistry with bacterial communities in streams in three Utah watersheds (Red Butte Creek, Provo River, and Logan River) across three seasons (Fall, Winter, and Spring).** Vectors represent positive correlations between environmental factors and a sample community composition. Location indicates position relative to man-made reservoirs and urban centers.

** Supplementary Figure 4.** **Redundancy analysis (RDA) plot relating stream major ion concentrations with bacterial communities in streams in three Utah watersheds (Red Butte Creek, Provo River, and Logan River) across three seasons (Fall, Winter, and Spring).** Vectors represent positive correlations between environmental factors and a sample community composition. Location indicates position relative to man-made reservoirs and urban centers.

**Supplementary Figure 5.** **Redundancy analysis (RDA) plot relating stream minor ions, trace elements, and water isotopes with bacterial communities in streams in three Utah watersheds (Red Butte Creek, Provo River, and Logan River) across three seasons (Fall, Winter, and Spring).** Vectors represent positive correlations between environmental factors and a sample community composition. Location indicates position relative to man-made reservoirs and urban centers.

**Supplementary Figure 6.** **Redundancy analysis (RDA) plot relating stream environmental variables and bacterial communities in streams in three Utah watersheds (Red Butte Creek, Provo River, and Logan River) across three seasons (Fall, Winter, and Spring).** Vectors represent positive correlations between environmental factors and a sample community composition. Location indicates position relative to man-made reservoirs and urban centers.
